# How might patient involvement in healthcare quality improvement efforts work—A realist literature review

**DOI:** 10.1111/hex.12900

**Published:** 2019-05-01

**Authors:** Carolina Bergerum, Johan Thor, Karin Josefsson, Maria Wolmesjö

**Affiliations:** ^1^ Faculty of Caring Science, Work Life and Social Welfare University of Borås Borås Sweden; ^2^ School of Health and Welfare, Jönköping Academy for Improvement of Health and Welfare Jönköping University Jönköping Sweden

**Keywords:** clinical microsystem, co‐design, co‐production, healthcare management, healthcare organization, patient involvement, quality improvement, realist review

## Abstract

**Introduction:**

This realist literature review, regarding active patient involvement in healthcare quality improvement (QI), seeks to identify possible mechanisms that contribute to success or failure. Furthermore, the paper outlines key considerations for organizing and supporting patient involvement in healthcare QI efforts.

**Methods:**

Two literature searches were performed. Altogether, 1204 articles from a healthcare context were screened, focusing on improvement efforts that involve patients, healthcare professionals and/or managers and leaders. Among these, 107 articles fulfilled the chosen study selection criteria and were further analysed. Eighteen articles underwent a full realist review. In the realist synthesis, context‐mechanism‐outcome configurations were articulated as middle‐range theories and organized thematically to generate a program theory on how active patient involvement in QI efforts might work.

**Results:**

The articles exhibited a diversity of patient involvement approaches at different levels of healthcare organizations. To be successful, organizations’ support of QI efforts that actively involved patients tailored the QI efforts to their context to achieve the desired outcomes, and involved the relevant microsystem members. Furthermore, it promoted interaction and partnership within the microsystem, and supported the behavioural change that follows.

**Conclusion:**

This realist synthesis generates a program theory for active patient involvement in QI efforts; active patient involvement can be a tool (resource), if tailored for interaction and partnership (reasoning), that leads to behaviour change (outcome) within healthcare QI efforts. The theory explains essential resource and reasoning mechanisms, and outcomes that together form guidance for healthcare organizations when managing active patient involvement in QI efforts.

## 1 INTRODUCTION

Patient involvement in health‐care improvement is attracting interest.^1^, ^2^, ^3^ Due to lived experiences of different health conditions and receiving health care, patients can contribute to health‐care improvement.^3^, ^4^, ^5^, ^6^, ^7^ Increasingly, health‐care professionals are expected to involve patients at different levels of health care, and health‐care organizations and their leaders are expected to support such efforts.^8^, ^9^, ^10^, ^11^ Societal focus on health‐care quality, patient safety and patients’ health‐care experiences, and growing rejection of paternalism further drives efforts to involve patients—the era of co‐production and co‐design.^4^, ^5^, ^7^, ^12^ In the literature, patient involvement has been described by many terms with diverse definitions—patient‐ or person‐centred care, patient or user participation and engagement, co‐creation, co‐design, co‐production, etc^3^, ^4^, ^5^, ^6^, ^7^, ^12^ Yet, there is no universally agreed‐upon definition of the different patient involvement concepts or what aspects should be fulfilled for each concept. Furthermore, there are few examples, and little knowledge, of how to organize for it. These limitations in the literature cause confusion for patients, health‐care professionals, managers and health‐care organizations.^3^, ^4^, ^5^, ^6^, ^7^

The science of quality improvement (QI) in health care concerns how to conduct QI and how to narrow the gap between current health‐care practice and the best possible practice.^13^, ^14^ It focuses on “what works” to improve quality and the best ways to capture and spread lessons learned to promote positive change. Therefore, it may inform the design, or re‐design, of complex health‐care services.^13^, ^14^, ^15^, ^16^, ^17^, ^18^ The present study rests on the premise that the health‐care system consists of clinical microsystems, which are nested in meso‐ and overarching macrosystems.^19^ Clinical microsystems are the smallest, functional units of a health‐care system where patients and health‐care professionals meet—for example an emergency room or a primary care centre. Microsystem interactions produce quality, safety and cost outcomes at the frontlines of health care. Macrosystem outcomes depend on the outcomes in the microsystems it harbours. Therefore, to improve and sustain quality in a health‐care system, key leverage points exist at the clinical microsystem level.^19^, ^20^ Considering the growing interest in active patient involvement in QI, where the patients hold the role as co‐creators,^21^ the uncertainty over how best to orchestrate such involvement, and what outcomes to expect on micro‐, meso‐, and macrosystem levels,^12^, ^22^ it is important to understand how approaches to patient involvement might work. The realist literature review approach aims to determine what works for whom, in what circumstances, in what respects and why.^23^, ^24^, ^25^

Guided by questions from a local hospital organization about how to involve patients in QI activities, we set out to review studies with active patient involvement in QI. We aimed to reveal how patient involvement in QI interventions might work in different contexts, to articulate guidance for health‐care organizations on managing active patient involvement in their QI efforts.

## 2 METHODS

### 2.1 Realist literature review framework

The realist literature review framework^23^, ^24^, ^25^, ^26^ seeks to identify and explain the interaction between context, mechanism and outcome, here regarding mechanisms for patient involvement in QI. With its philosophical basis in realism, the framework was developed for complex social interventions. It is a systematic, theory‐driven interpretative technique. The approach determines what works, how, for whom, to what extent and under what conditions, expressed as “program theory.” 23, 24, 25, 26, 27 It was developed to make sense of heterogeneous evidence about complex interventions applied in diverse contexts, and focuses on how different contexts (C) interact with different mechanisms (M) to make particular outcomes (O) more or less likely. This is expressed in “C + M = O” formulas. Consequently, a realist review proposes general recommendations in the following format: “In situations (X), complex intervention (Y), modified in this way and taking account of these circumstances, may be appropriate” to yield these outcomes (O).^26^

### 2.2 Search strategy

Due to qualitative research, this study has been presented at seminars for colleagues from different disciplines and has evolved accordingly. Based on an initial search and review, we focused on patients’ active involvement in QI efforts, guided by feedback from colleagues, and undertook a complementary second literature search. Both search strategies were developed in collaboration with a university librarian and included the following electronic databases: the Web of Science (Core Collection), Scopus, Cinahl and PubMed. Authors and stakeholders were interested in the field's recent developments, and we, therefore, limited the search to articles published from 2011 forward.

The first, broader search, which included articles published from January 2011 until February 2016, combined the following terms and keywords: quality improvement, healthcare, service, involvement, patients, next of kin, professionals, managers and leaders. This also included literature that addressed health‐care improvement more broadly, such as value‐based care and the application of clinical microsystem thinking. The second search, covering January 2011 to September 2017, focused, more specifically, on active patient involvement in QI. Terms and keywords included: user involvement, quality improvement, healthcare, service, patients, next of kin, professionals, managers and leaders. Furthermore, this search included literature that addressed the words: patient, participation, involvement, collaboration and service design. The Boolean terms “AND,” “OR” and “NEAR” were used to find the words’ intersections. The search approaches were modified as necessary to fit each database. Altogether, the two searches yielded 1204 articles.

### 2.3 Study selection

Each article's title, abstract and subject headings were screened according to the following criteria:

- Publication type—original peer‐reviewed articles, published in English.
- Setting—hospital care, inpatient or outpatient hospital care; single speciality setting, multiple specialities in collaboration and primary health care.
- Population—patients, health‐care professionals, managers and leaders.
- Interventions—clinical QI work that involved patients, families, next of kin, health‐care professionals and/or managers and leaders.
- Outcome reporting—empirical, clinical QI efforts, with patient health outcomes, system performance outcomes (care and/or costs), and/or professional development as the primary outcome measure.

**Figure 1:**
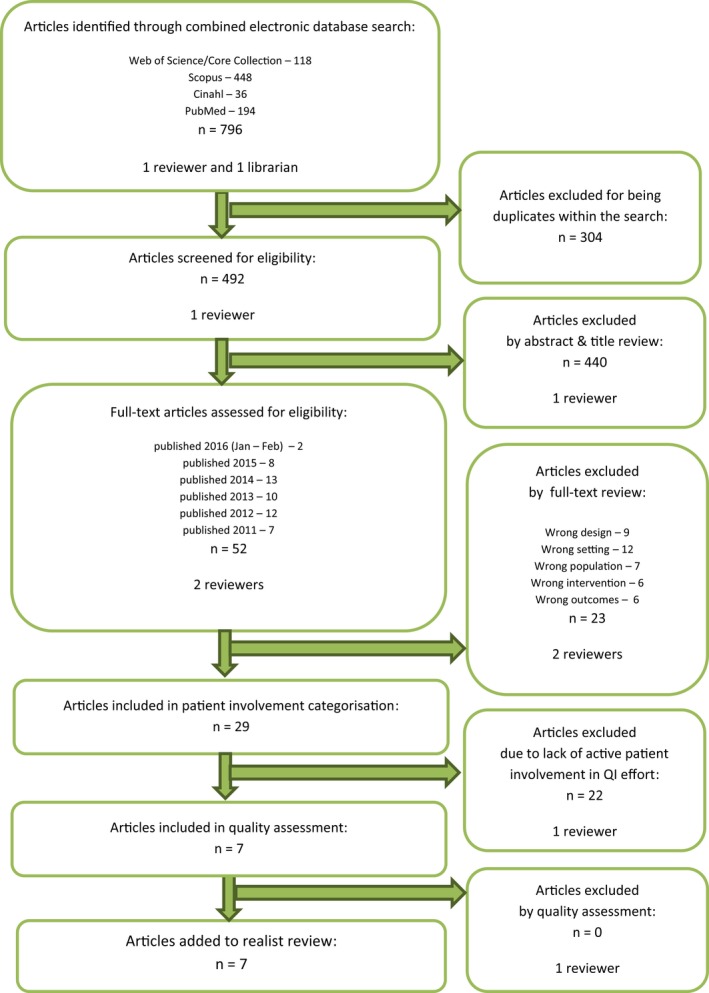
First article selection flow diagram

**Figure 2:**
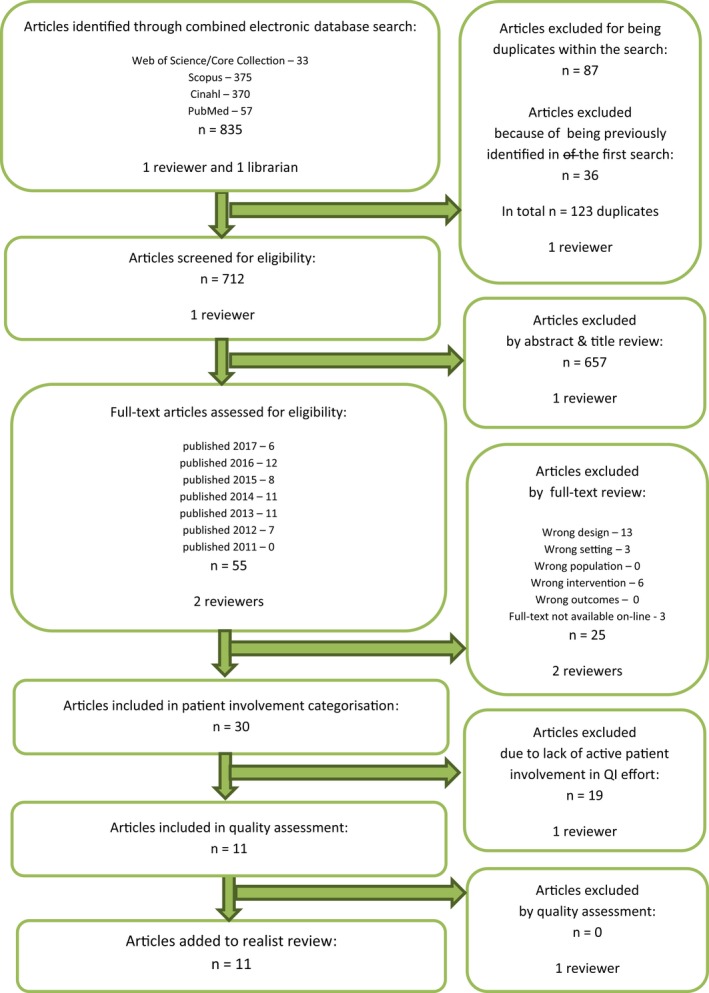
Second article selection flow diagram

After this first screening, two of the study's authors independently reviewed the remaining 107 articles, in full text, against the above selection criteria. Discrepancies were resolved by consensus, and reasons for exclusion were documented for each article. This step yielded 59 articles, many of which concerned QI efforts to develop patient involvement in health care, without patients actively taking part in those QI efforts. We, therefore, selected the subgroup of articles with active patient involvement, resulting in 18 articles. The study selection procedures are displayed in the article selection flow diagrams (Figures 1 and 2).

### 2.4 Data collection

A data collection protocol was developed by two of this study's authors, and, in the data extraction procedure, they compared their respective data collections. The protocol is available in Table S1.

### 2.5 Quality assessment

To assess articles’ methodological quality, two authors developed criteria based on the Standards for Quality Improvement Reporting Excellence (SQUIRE) guidelines.^28^ The 26 criteria concern the rationale, specific aims, context, intervention(s), study of the intervention(s) measures, analysis, ethical considerations, results, interpretation, limitations, conclusions and funding. A methodological quality score was developed as a three‐point scale, ranging from “poor,” to “fair” to “good.” Each article was given its methodological quality score by simply counting the number of criteria satisfied. For an article to be scored as “good,” at least 20 criteria had to be fulfilled. No article was excluded at this stage, so all 18 were brought into the realist review procedure.

### 2.6 The realist synthesis procedure

To reflect the articles’ heterogeneity, they were categorized by the organizational level of their patient involvement approach^3^ and by the complexity of problems and interventions^29^ (outlined in the findings section). The literature was approached to identify mechanisms that explain why health‐care QI involving patients might, or might not, work. The synthesis involved comparing findings regarding the review questions across health‐care settings to articulate the conditions that support or hinder active patient involvement.^23^, ^25^

The review questions were:

- What are the key mechanisms influencing or driving the QI effort?
- What contextual factors have the most impact?
- How might health‐care organizations support active patient involvement in QI?

The questions were viewed from the perspectives of patients, health‐care professionals, managers and leaders.^19^, ^20^, ^24^, ^26^ To complement data on the study characteristics outlined above, we identified each article's theoretical contribution—that is “how” patient involvement in QI works, “for whom,” “to what extent” and “under what conditions.” We extracted illustrative quotes and summarized, in a spreadsheet, each article's contents relevant to the review questions.

**Figure 3:**
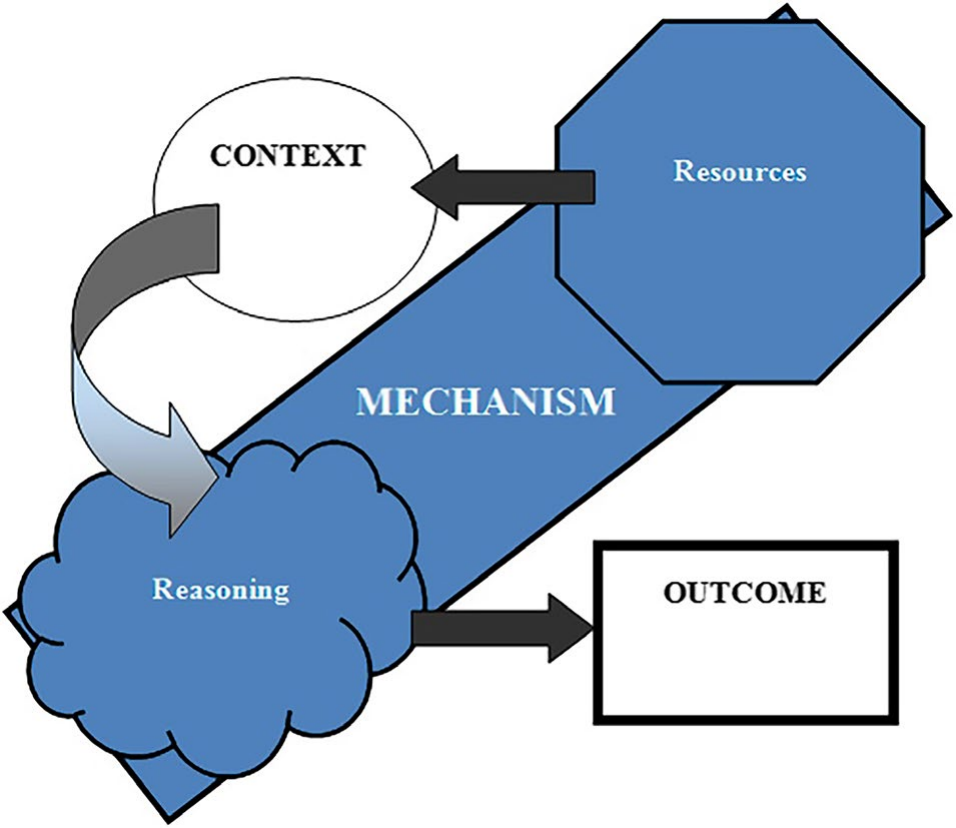
The context‐mechanism‐outcome configuration framework, distinguishing the resource and reasoning aspects of mechanism^30^ (reprinted with permission)

In practice, the articles were read several times to gain a general overview. Each article was then reviewed individually for C‐M‐O configurations (CMOc). In CMOc, the mechanisms explain what an intervention—for example patient involvement in QI—triggers in a given context that makes things happen to produce observable outcomes. Drawing on methodological guidance to distinguish the context from the mechanism,^30^ we split the “C + M = O” formula's mechanism component into “mechanism resource” (the component introduced in a context) and “mechanism reasoning” (stakeholders’ volition), yielding the formula “M resource + C → M reasoning = O” (Figure 3).

The synthesis involved identifying and articulating “middle‐range theories,” that is theoretical explanations of CMOc.^24^ “Middle‐range theories” explain examples of success, failure and the variations in between. They involve abstraction but are concrete enough to permit empirical testing. The “middle‐range theories” emerged in the process of identifying CMOc relevant to the review questions, mapping patterns of findings, and sense‐making. They were then organized thematically and expressed as theories.^31^, ^32^ This procedure is demonstrated in Supplement 2, and the full procedure is available upon request.

The program theory^31^, ^32^ was generated in an iterative procedure. Several methods were used for this, such as brain‐storming, following references of references, browsing grey literature^23^—including internal reports, national policy documents and websites—discussing within the research team and with other researchers, and with local health‐care improvement facilitators. Thus, synthesizing the evidence, the theories were articulated, and the authors drew the study's conclusions. In line with the realist literature review process,^23^, ^24^ this yielded a program theory on how patient involvement in QI interventions might work in different contexts, presented below.

### 2.7 Findings

The search strategies yielded 1,204 articles total. In assessing the methodological quality of the 18 articles included in the review, nine articles scored > 20 (ranging 20 ‐ 23) for “good” quality, 33, 34, 35, 36, 37, 38, 39, 40, 41 and nine scored “fair” (ranging 11‐19).^42^, ^43^, ^44^, ^45^, ^46^, ^47^, ^48^, ^49^, ^50^ Weaknesses were noted in several studies. For example, methods employed for assessing data completeness and accuracy, and for understanding variation within the data, were not always described. Ethical considerations were not declared in several studies. Unintended consequences and details about missing data were not always discussed, and efforts made to minimize and adjust for limitations were not consistently declared. Nevertheless, since all articles exhibited at least fair quality, they were all equally considered in the analysis.

### 2.8 Description of studies

We categorized patient involvement concepts from the 18 review studies according to Gustavsson's organizational levels of patient involvement^3^, ^4^, ^5^, ^51^, ^52^, ^53^, ^54^, ^55^, ^56^, ^57^, ^58^, ^59^, ^60^, ^61^, ^62^ and to the Glouberman and Zimmerman^29^ complexity typology (Tables 1 and 2).

**Table 1:** Studies in the review (n = 18) categorized by approach to patient involvement in QI and by organizational level of application, as proposed by Gustavsson^3^

Organizational level(s) of patient involvement in QI	Patient involvement approach	Studies (n = 18)
Individual level (n = 2)	Patient‐centred care^51^, ^52^ (n = 1)	Benzo et al (2013)^42^
	Family‐centred care^53^, ^54^, ^55^ (n = 0)	
	Person‐centred care^56^, ^57^ (n = 0)	
	Patient participation^58^ (n = 0)	
	Co‐creation^59^ (n = 1)	Olsson et al (2014)^33^
Individual and group level (n = 2)	Co‐production^60^ (n = 2)	Robben et al (2012)^34^ Worswick et al (2015)^43^
Individual, group, governance and management, and societal level (*n* = 14)	Patient engagement^61^, ^62^ (n = 4)	Armstrong et al (2013)^44^ Lachman et al (2015)^35^ Pittens et al (2015)^36^ Rise et al (2014)^37^
	Co‐design^4^, ^5^ (n = 10)	Boaz et al (2016)^38^ Boivin et al (2014)^39^ de Souza et al (2017)^45^ Gustavsson (2014)^40^ Lavoie‐Tremblay et al (2014)^46^ Locock et al (2014)^47^ Morrison & Dearden (2013)^48^ Noergaard et al (2016)^41^ Tollyfield (2014)^49^ Tsianakas et al (2012)^50^

Note

Studies concerned one, two or all four of these organizational levels: (1) The individual level (activities concerning an individual's own care).^51^, ^52^, ^53^, ^54^, ^55^, ^56^, ^57^, ^58^, ^59^, ^60^ (2) The group level (service delivery activities).^60^ (3) The governance and management level (being part of leadership and management).^61^, ^62^ (4) The societal level (co‐researching, policy‐making).^61^, ^62^

**Table 2:** Studies (n = 18) cross‐tabulated by the complexity of health‐care problems and of interventions to address them: simple, complicated and complex^29^

Health‐care problem	Intervention		
	Simple (n = 0)	Complicated (n = 2)	Complex (n = 16)
Simple (n = 0)			
Complicated (n = 1)		Lachman et al (2015)^35^	
Complex (n = 17)		Boivin et al (2014)^39^	Armstrong et al (2013)^44^ Benzo et al (2013)^42^ Boaz et al (2016)^38^ de Souza et al (2017)^45^ Gustavsson (2014)^40^ Lavoie‐Tremblay et al (2014)^46^ Locock et al (2014)^47^ Morrison & Dearden (2013)^48^ Noergaard et al (2016)^41^ Olsson et al (2014)^33^ Pittens et al (2015)^36^ Rise et al (2014)^37^ Robben et al (2012)^34^ Tollyfield (2014)^49^ Tsianakas et al (2012)^50^ Worswick et al (2015)^43^

#### 2.8.1 Three theories for managing patient involvement

Reviewing the 18 articles, we derived 36 sets of CMOc, some of them interrelated (exemplified in Table S2). Thematically synthesizing the “middle‐range theories” based on CMOc, three theories^31^, ^32^ emerged. They indicate how QI might work in health‐care organizations, by (a) tailoring patient involvement to the various QI efforts and contexts, (b) supporting interaction and partnership within each microsystem's QI effort and (c) supporting the behavioural change that follows from QI efforts involving users, at all organizational levels.

### 2.9 Synthesis of results

#### 2.9.1 Tailoring

Involving members of the relevant microsystems—the small, functional units where patients and health‐care professionals meet—influences and promotes QI efforts at all organizational levels. Enabling patients, and/or their next of kin, to share their individual goals and concerns with health‐care professionals in a direct, real‐time way within the microsystem supports their involvement. All studies included in the review described such person‐specific and individualized interventions, where patients were actively involved and put in the lead—enabled to prioritize their needs and participate in an informed way through, for example, self‐management training, outpatient health‐care visits, patient safety issues or co‐design QI efforts.^33^, ^35^, ^36^, ^38^, ^40^, ^41^, ^42^, ^43^, ^44^, ^45^, ^46^, ^49^, ^50^

To reach a specific target group, for example immigrant women, involvement of other key actors in the QI effort can be helpful. In one study, the involvement of local doulas who shared immigrant women's cultural background and mother tongue indirectly supported patient involvement in cervical cancer screening. They were involved in the identification of barriers and planning, and the execution of the QI effort and were able to encourage the immigrant women on their own terms. As a result, the number of cervical cancer screening tests increased by an average of 40% during the intervention period.^33^

An iterative QI process, tailored to a microsystem's circumstances and priorities and to research evidence, can also strengthen the responsiveness mechanism related to an intervention.^34^, ^36^, ^45^ For example, a co‐design QI approach, where patients and health‐care professionals collaborated, focused on efforts that met *both* patients’ *and* health‐care professionals’ needs and priorities. In an outpatient rheumatology service, “the process [allows] patients to directly contribute to shaping the services they receive long‐term and realizing their opinions were of value to clinical staff and hospital management.” 45 QI priorities within a microsystem can be identified when patients and health‐care professionals exchange stories and experiences in face‐to‐face meetings, co‐design discussions and jointly prioritize improvement efforts. Such an approach indicates the importance of prioritizing and conducting QI, and, in turn, this reasoning may promote QI effort sustainability.^39^, ^40^, ^41^, ^46^, ^47^, ^48^, ^49^, ^50^

Tailoring microsystem involvement demands organizational understanding of the resource and reasoning mechanisms involved. One case,^37^ studying user involvement at several organizational levels suggests that to consider microsystem involvement valuable and recognize its effects, stakeholders benefitted from experiencing it in practice. The intervention concerned implementing a plan to enhance user involvement in a mental health hospital, and the results illustrate that the closer the personal involvement in the implementation process, the greater the reported experience of success. Participants who experienced the greatest success were those who had actively worked on the implementation, whereas the peripherally involved managers and health‐care professionals believed the initiative had limited impact. The synthesis indicates that a distant organizational relationship to patient involvement may prevent understanding of immediate and implicit advantages participants experience in the microsystem. From a macrosystem perspective, user involvement then risks being reasoned away as only adding workload without returning any value, and it may, therefore, be poorly supported. However, this can be prevented if an organization's leadership address barriers related to organizational culture, entrusts the QI decision‐power to the microsystem involved and recognizes the improvements that are accomplished.^37^, ^39^, ^45^

While many problems and interventions in health care are complicated or complex, successful QI interventions can also consist of simple and basic tools.^35^, ^48^ For example, in a project engaging patients and families to report harm, introducing a simple, real‐time bedside tool triggered positive change in a ward's overall safety culture. By offering direct patient feedback, previously unrecognized areas of harm were detected, and health‐care professionals’ reporting of harm increased.^35^ Thus, facilitating such simple and low‐cost intervention tools, and realizing their impact on individual patient involvement, can lead to further reasoning mechanisms and behaviour outcomes at the microsystem level.^35^, ^48^

Active patient involvement in health‐care QI requires continuous, organizational preparation and facilitation.^33^, ^34^, ^35^, ^36^, ^37^, ^38^, ^39^, ^40^, ^41^, ^42^, ^43^, ^44^, ^45^, ^46^, ^47^, ^48^, ^49^, ^50^ Clarification of the rationale to all actors, the QI effort's purpose, as well as participant roles and responsibilities, must be outlined from the start.^44^ To trigger discussion and reasoning within the microsystem, preparing a comfortable physical environment for meetings and establishing effective communication channels are two of the practical conditions to be satisfied. Discussion and reasoning are also triggered by, for example, using stories and experiences.^38^, ^44^, ^47^, ^48^ Equally involving patients and health‐care professionals may be complex and challenging, due, for example, to patient frailty or other conditions which limit stakeholders’ ability to participate, or when scientific evidence and the locally expressed microsystem needs point in different directions.^34^ Therefore, facilitation must be flexible and sensitive to each QI effort's context, both individually and at the group level.^34^, ^38^, ^44^ In a successful example with cancer patients,^50^ the carefully tailored intervention led to a joint awareness of the connection between patients’ health‐care experiences and microsystem QI efforts, which furthermore led to shared responsibility and empowerment within the microsystem. In multiple studies, such shared responsibility and empowerment reasoning promoted the QI intervention development.^33^, ^34^, ^35^, ^39^, ^40^, ^41^, ^42^, ^43^, ^47^, ^48^, ^49^, ^50^

Theory 1: Tailoring patient involvement (resource and reasoning) to each QI effort (context) may lead to interaction and partnership within the microsystem (outcome).

#### 2.9.2 Interaction and partnership

In studies involving co‐design interventions,^38^, ^39^, ^40^, ^41^, ^45^, ^46^, ^47^, ^48^, ^49^, ^50^ patients and health‐care professionals jointly identified and prioritized meaningful QI efforts, based on mutual understanding gained in partnership. This suggests that, to promote active patient involvement at all levels in a health‐care organization, efforts must start at the microsystem level, where patients prioritize their needs actively and in an informed way. Interaction and partnership between patients and health‐care professionals is an important resource mechanism for patient involvement in QI projects. For example, well‐facilitated face‐to‐face meetings, encouraging participants to listen to each other and to reflect, promote development of these relationships and co‐operation methods.^38^, ^39^, ^40^, ^41^, ^45^, ^46^, ^47^, ^48^, ^49^, ^50^

No matter what type of intervention applied, what context involved or what organizational level at hand, QI efforts seem more successful when patients are invited to share their individual concerns, and when health‐care professionals respond to them relevantly.^33^, ^34^, ^35^, ^36^, ^37^, ^38^, ^39^, ^40^, ^41^, ^42^, ^43^, ^44^, ^45^, ^46^, ^47^, ^48^, ^49^, ^50^ Therefore, the macrosystem facilitation must be sensitive and tailored to each QI effort and its particular organizational context.^33^, ^34^, ^38^, ^42^, ^43^, ^44^ At the individual level, simple communication tools, such as emotion maps, patient stories or films to facilitate discussion in co‐design projects, support responsive reasoning and help both patients and health‐care professionals interact in QI efforts.^35^, ^41^, ^42^, ^48^, ^49^, ^50^ Basic and low‐cost tools may also be the most suitable and applicable for QI practice because of their simple and user‐friendly support for interaction. Such person‐centred tools for immediate feedback promote sharing of goals and responsibilities between patients and health‐care professionals. The previously mentioned study that developed a tool for patients and families to report harm argues for this reasoning.^35^ Furthermore, the study suggests such tools contribute to increased ward safety culture by raising awareness and helping health‐care professionals know what is happening in real time.

Interaction itself provides an important feedback resource for patients and health‐care professionals.^36^, ^37^, ^38^, ^39^, ^40^, ^42^, ^43^, ^46^, ^47^, ^49^, ^50^ A self‐management intervention using Motivational Interviewing (MI) skills for patients with chronic obstructive pulmonary disease exemplifies this. Patients were involved in the intervention's development and application. The research evaluation showed that the MI approach supported health‐care professionals in having a more personalized and collaborative approach, which in return was recognized and valued by the patients. Following this, increased patient engagement and emerging commitment to self‐management was reported. Thus, behaviour change was seen among both patients and health‐care professionals.^42^ An example of the opposite resource and reasoning is found in a QI effort involving patients in gynaecological guideline development. In this intervention, no direct interaction between patients and health‐care professionals was facilitated. From the beginning, patient input was limited to one part of the project, but, as the project developed, it also influenced other parts. Lacking integration of patients and health‐care professionals in participatory activities may have prevented mechanisms of mutual learning and evolution within the microsystem, thus limiting the developed guideline's relevance and quality.^36^

Theory 2: Supporting interaction and partnership within the microsystem (resource and reasoning) of each specific QI effort (context) may lead to behavioural change (outcome).

#### 2.9.3 Behavioural change

Several of the included studies refer to organizational changes and to the attitudinal changes among patients and health‐care professionals that may follow from microsystem QI efforts.^35^, ^37^, ^38^, ^41^, ^43^, ^45^, ^46^, ^48^ The reasoning mechanism triggered may be a greater receptivity for change that influences both professional and personal behaviours and attitudes at the individual level, as described in an Experience‐Based Co‐Design (EBCD) context.^38^ This reasoning mechanism, in which patient involvement is enabled at each individual's choice of level, may increase mutual understanding. In turn, this mutual understanding may lead to increased motivation for change and, therefore, inspire wider organizational and attitudinal changes.

Patients, health‐care professionals and organizational leaders affect each other's behaviours. In the harm self‐reporting intervention,^41^ when patient‐reporting of incidents was introduced, the number of incidents reported by health‐care professionals also increased. A probable explanation lies within the behavioural change that seems to follow from involving immediate feedback within a QI effort. Therefore, successful organizational support may lie in facilitating respectful and equal contexts^41^ where, for example, a common language enabling common understanding between patients and health‐care professionals is promoted.^48^ In line with this reasoning, some obstacles related to individual commitment and organizational culture are noted in a study describing a service design intervention to improve rheumatology outpatients’ experiences.^45^ The authors discuss the possibility of these barriers lying in health‐care professionals’ and managers’ beliefs that patients cannot make effective contributions—as well as the perceived threat of “losing face” by sharing organizational shortcomings and difficulties.

To support the emerging co‐learning within the microsystem during QI efforts, organizational support is suggested to be an on‐going process.^43^ Additionally, a reasoning mechanism to support QI efforts can be for the organization to realize its peripheral involvement in the process and trust the microsystem with decision‐power. In practice, the organizational macro‐level can facilitate QI efforts by recognizing and acknowledging the behavioural changes that follow from active patient involvement.^37^ In turn, the mutual agreement achieved within the QI efforts will ensure the prioritizing of feasible interventions that matter the most to patients and lead to sustainable changes for patients and health‐care professionals.^41^

Theory 3: Support (resource and reasoning mechanism) the behavioural change (outcome) that follows from QI efforts involving patients (context) at all organizational levels.

#### 2.9.4 The program theory for effective patient involvement

**Figure 4:**
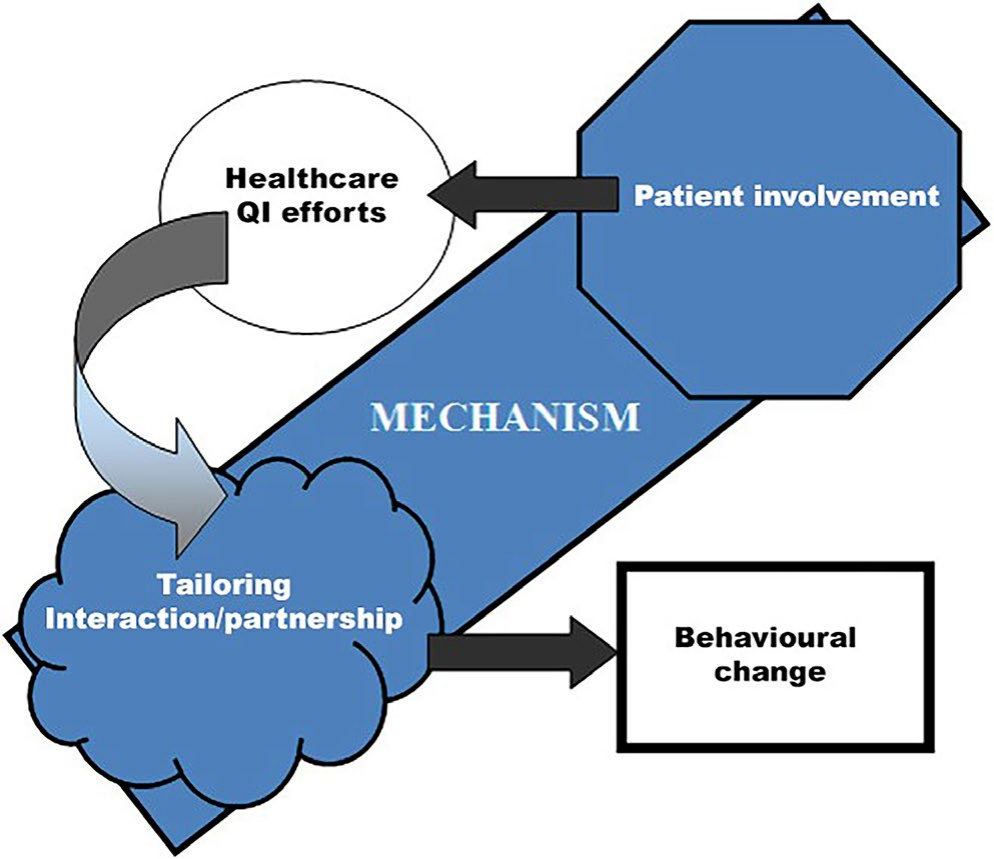
The program theory illustrated in a context‐mechanism‐outcome configuration.^30^ Patient involvement as a tool (resource), tailored for interaction and partnership (reasoning), leading to behaviour change (outcome) within health‐care QI efforts (context)

In the synthesis procedure, a program theory was generated. It became clear that active patient involvement can be a tool (resource), if tailored for interaction and partnership (reasoning), that leads to behaviour change (outcome) within health‐care QI efforts (context) (Figure 4).

## 3 DISCUSSION

This realist synthesis suggests a program theory to guide health‐care organizations when involving patients in improving health‐care quality; tailor patient involvement to various QI efforts and contexts, support interaction and partnership within each QI effort, and support behavioural changes that follow QI efforts involving patients—at all organizational levels. These findings may seem self‐evident; however, the gap between health‐care policy and practice remains, with barriers and uncertainty concerning how to best involve patients on different organizational levels.^3^, ^12^, ^22^, ^63^, ^64^, ^65^, ^66^, ^67^ Patient involvement includes many aspects and issues, and the term is not clearly understood, by either patients or healthcare professionals.^68^ Additionally, the empirical literature search reveals a relative lack of evidence. Many evaluations are not published as peer‐reviewed articles, but as internal reports, national policy documents and websites. This indicates that informal QI knowledge and theory exist within healthcare organizations. It would be helpful if this knowledge was made scientifically explicit^69^, ^70^, ^71^ to reveal “what works” and to indicate the best ways of harnessing and spreading lessons learned from efforts involving patients in QI.

Nevertheless, this study did find examples, across all four organizational patient involvement levels,^3^ in which the shared involvement of relevant microsystem members promoted QI efforts. Members of clinical microsystems where patients, and if relevant other adequate actors,^33^ are included can actively contribute to healthcare re‐design and improvement.^19^, ^20^, ^33^, ^34^, ^35^, ^36^, ^37^, ^38^, ^39^, ^40^, ^41^, ^42^, ^43^, ^44^, ^45^, ^46^, ^47^, ^48^, ^49^, ^50^, ^72^ Additionally, the level of involvement influences both explicit and implicit outcomes.^22^, ^37^ To improve and sustain healthcare quality, healthcare professionals should be supported by their organization in partnering with the patients in their clinical microsystem.^19^, ^20^, ^63^, ^64^ Pointing in the same direction, a study from the National Institute for Health Research (NIHR) explored the links between patients’ experiences of healthcare and healthcare professionals’ motivation and well‐being.^64^ It found that, in a setting where healthcare professionals’ well‐being is good, patient experience also is generally good. This indicates that patients and health‐care professionals influence each other positively when given the opportunity.^33^, ^34^, ^35^, ^36^, ^37^, ^38^, ^39^, ^40^, ^41^, ^42^, ^43^, ^44^, ^45^, ^46^, ^47^, ^48^, ^49^, ^50^, ^61^, ^62^

Fitting the patient involvement resource to each QI effort's problems and contexts is a complex, but inevitable and critical, undertaking for health‐care organizations. Patient involvement is directed by guidelines and regulations.^8^, ^9^, ^10^ However, in practice, it is also influenced by resource (the intervention introduced) and reasoning (the microsystem members’ volition) mechanisms interacting with each context. These mechanisms affect each intervention's progression and lead to a range of heterogeneous outcomes,^29^, ^30^, ^32^ adding to the complexity challenge. In our study, we have identified tailoring, interaction and partnership, and behavioural change as resource and reasoning mechanisms—and outcomes—for health‐care organizations to be aware of when managing QI efforts involving patients. Based on this, we propose that it can be clarifying for health‐care organizations to characterize their health‐care problems and interventions as being simple, complicated or complex,^29^ and to simultaneously consider the different health‐care organization levels for patient involvement^3^ when planning and designing QI efforts.

The literature indicates many barriers for organizations to identify and consider when managing QI efforts. Barriers may concern health‐care system financing, competing organizational changes and the work environment—such as time constraints, staffing, routines, educational skills and the existing attitudes and culture.^43^, ^49^, ^64^, ^65^, ^72^ When validating prior work,^3^, ^4^, ^5^, ^6^, ^7^ we found that organizational support for interaction and partnership within the microsystem is an essential resource and reasoning mechanism for patient involvement in QI efforts. As active patient involvement is a relatively new and insufficiently understood resource, it requires thoughtful management to ensure processes are meaningful and facilitation is flexible and sensitive to each intervention's requirements, individual preferences, existing power relations and context.^3^, ^5^, ^7^, ^38^, ^43^, ^44^, ^47^, ^49^, ^50^, ^63^

The diversity of patient involvement concepts and definitions^1^, ^2^, ^3^, ^51^, ^52^ implies a lack of agreement on, and perhaps understanding of, the nature of patient involvement itself, and of how to strengthen and harness it in health‐care QI. The impact of patients’ and health‐care professionals’ involvement in QI efforts is a complex issue that is poorly addressed in both health‐care practice and research.^65^ Perhaps the largest barriers to knowledge and understanding lie within existing attitudes and culture.^43^, ^45^, ^49^, ^64^, ^65^, ^72^ Many stakeholders with diverse expectations are involved (patient representatives, health‐care professionals, policy makers, funders, researchers), and the gap between health‐care policy and practice may contribute to the lack of clarity.^65^, ^66^, ^67^, ^73^ Therefore, patient involvement runs the risk of becoming tokenistic,^65^, ^73^ which may limit synergies between co‐production and value creation (or may even cause value destruction) for patients and health‐care organizations.^21^, ^60^

Despite limited evidence, patient involvement is reasoned to be a probable “tool” for cultural change because it impacts attitudes, values and assumptions within the microsystem.^66^ Our study also suggests patient involvement should be a tool (resource), if tailored for interaction and partnership (reasoning), leading to behaviour change (outcome) within health‐care QI efforts (context) (Figure 4).

Health‐care organizations are responsible for closing the gap between health‐care policy and practice. Besides facilitating QI efforts at the microsystem level, and supporting interaction and partnership within the microsystem, this review has revealed the behavioural change that follows from involving patients in QI efforts. A major accomplishment lies within recognizing and supporting this behavioural change. Thoughtful and proper evaluation and feedback is needed—for example by developing and monitoring patient‐centred outcomes^74^ and evaluating health‐care professionals’ motivation and well‐being. It is an on‐going pursuit of organizational behaviour change^43^, ^64^, ^65^, ^67^, ^73^ in the era of co‐production and co‐design.^4^, ^5^, ^7^, ^12^ Further research in this area is warranted.^1^, ^2^, ^22^, ^67^

### 3.1 Methodological considerations

There are limits on what a realist review can cover.^25^ Although guided by a professional librarian, this study's two searches failed to include all terms and key words available, which arguably reflects the obscure and on‐going creation of terms related to patient involvement. Future searches would benefit from more consistent key words and MeSH terms.

Narrowing the subgroup of articles to active patient involvement in QI efforts reduced the literature set for review. Several successful involvement efforts, concerning, for example, family‐centred care, were excluded. Because of this and the heterogeneity of studies, we could not develop recommendations following the format “In situations (X), complex intervention (Y), modified in this way and taking account of these circumstances, may be appropriate.”^23^, ^26^ Furthermore, the theories proposed here are limited by what was expressed in the included studies, several of which exhibited minor methodological weaknesses. Nevertheless, reading documents drawn from reference lists and additional grey literature,^23^ while dialoguing with other researchers and health‐care improvement facilitators, helped us refine the results. However, due to these limitations, the findings should be interpreted cautiously, and the field will benefit from further research to expand this topic.

The realist review process requires flexibility and an ability to handle complexity, but it can reward reviewers and readers with pragmatic and applicable conclusions.^75^ Splitting the mechanism component of the “C + M = O” formula into resource and reasoning^25^ helps distinguish the context from the mechanism and, therefore, aids in understanding the difference between the resources (provided by the intervention) and how participants’ reasoning is changed in a particular context. For each theory, there are “middle‐range theories” articulating the mechanisms at hand. The studies we reviewed did not include any actual failures, but they did include examples of interventions that experienced obstacles. The realist review process enabled us to integrate these important study results.

Finally, the realist review approach emphasizes human means of reasoning and action, linking information in the studies on interventions’ resources to the outcomes achieved while considering the influence of context.^23^, ^24^, ^30^, ^32^ In health‐care QI efforts, resources, reasoning and the local context all matter. The shift towards co‐production and co‐design^12^ further emphasizes the importance of this. Therefore, health‐care organizations benefit from realist approaches to generating knowledge about how patient involvement might work, how, for whom, to what extent and under what conditions. This realist literature review does not claim to yield a final program theory, but it has identified and mapped out a program theory to be tested, refined and evaluated in practice and future studies. We, therefore, conclude that the results add to existing knowledge and can guide stakeholders in health‐care organizations and microsystems.^32^, ^71^, ^76^ Furthermore, studying organizations in other sectors that have successfully involved users would enable a deeper understanding of how health‐care organizations can involve patients in QI efforts ever more successfully.

## 4 CONCLUSION

This realist synthesis identifies three interdependent theories to guide health‐care organizations when involving patients in improving health‐care quality: tailoring, interaction and partnership, and behavioural change. They can be considered resource and reasoning mechanisms, as well as outcomes essential for QI efforts. Together, they form a program theory and guidance for health‐care organizations in managing active patient involvement in QI efforts; active patient involvement can be a tool (resource), if tailored for interaction and partnership (reasoning), that leads to behaviour change (outcome) within health‐care QI efforts. In healthcare co‐production and co‐design, resources, reasoning and the local context are all important. To further refine and develop a more nuanced and powerful program theory, research on how it works on different organizational levels, and from different stakeholder's perspectives, is required.
