# Supplementary material for: How might patient involvement in healthcare quality improvement efforts work—A realist literature review
Source: Health Expect. 2019 May 1;22(5):952–64. doi: 10.1111/hex.12900 (PMC6803394; doi:10.1111/hex.12900)
Supplement: Supplementary file 1 [file HEX-22-952-s001.docx]

**Supplement 1.** Table. Data collection protocol. 2^nd^ review.

| Article  Author  Title  Publication year  Country | **Study design** | **Settings**  – hospital care, in-patient or out-patient hospital care; single speciality setting, multiple specialities in collaboration; primary healthcare | **Population**  - patients, healthcare professionals, managers and leaders | **Interventions**  - clinical quality improvement work that engages patients, families, next of kin, healthcare professionals and or managers and leaders | **Outcome reporting of empirical clinical improvement work**   - patient outcomes (health) - system performance outcomes (care, economy) - professional development |
| --- | --- | --- | --- | --- | --- |
| Armstrong  Optimizing patient involvement in quality improvement  2013  UK | Case study  Ethnographic approach  Data analysis based on the constant comparative method | In-patient hospital care  Primary healthcare  Multidisciplinary teams  Three QI projects  1. lung cancer  2. aneurysm  3. kidney  were drawn from 11 projects participating in Closing the Gap through Clinical Communities (CTGTCC), a programme funded by the Health Foundation (THF) (a charitable foundation) | Patients  Healthcare professionals  Managers and leaders  12 weeks of non-participant observations    1. lung cancer (25,5 days)  2. aneurysm (21,5 days)  3. kidney (16,5 days)  Documents  126 in-depth interviews | Lung cancer   - charity representatives - patient experience data from questionnaires   Aneurysm   - patient representative on the project team - local patient representatives - patient focus groups   Kidney   - a ‘patients’ group’ - patient representative on the project team | Involvement – 3 different models evolved, influenced by each project context  Identification of strategies for patient involvement   - clarity on the rationale for patient involvement - identifying the right model to achieve the desired outcomes - clear roles and responsibilities for patients - involvement that is meaningful   Patients as a ‘technology of persuasion’  Patients as ‘knowledge brokers’  Conclusion: Patient involvement in quality improvement work needs careful management to realise its full potential |
| Benzo  Development and feasibility of a self-management intervention for chronic obstructive pulmonary disease delivered with motivational interviewing strategies  2013  USA | Pilot study to refine intervention  Mixed methods   - fidelity to protocol - qualitative feedback (content analysis) - effect on quality of life   Refined intervention was delivered as part a prospective randomised study | Hospital care  Chronic obstructive pulmonary disease (COPD) | Pilot study:  semi-structured interviews with 11 patients hospitalised for a COPD exacerbation  Refined intervention:  544 encounters in 44 patients randomised to the treatment arm of the study | A self-management program using Motivational Interviewing skills, aiming to increase engagement and commitment in severe COPD patients   - healthcare professionals trained in MI - weekly sessions for patients over 8 weeks   The study intervention merges both self-management education for patients and MI for healthcare professionals | Positive changes in the Chronic Respiratory Questionnaire (CRQ)   - dyspnaea - fatigue - emotional - mastery   High completion of scheduled sessions  Increased patient acceptability and satisfaction  No harm  Increassed self management  Increased MI  Positive perceptions of the patient/healthcare professional relationship  Personal approach valued – fosters collaboration  Conclusion: A self-management intervention, that includes motivational interviewing as the way if guiding patient into behavior change, is feasible in severe COPD and may increase patient engagement and commitment to self-management |
| Boaz  What patients do and their impact on implementation: An ethnographic study of participatory quality improvement projects in English acute hospitals  2016  UK | A cross-case ethnographic evaluation and comparison  Framework analysis (qualitative and quantitative data)  Observations  Interviews  Questionnaires  Reflective diaries  Service improvement logs  Documents | Hospital care  4 projects in two clinical pathways   - intensive care units (2 projects) - lung cancer units (2 projects) | 63 patients  Carers  Healthcare professionals  In total 14 co-design groups | Experience-based Co-design (EBCD) | Different roles adopted by patients and carers in relation to implementation   - participation as co-designers - ongoing patient engagement as ‘experts by experience’   Often small-scale QI changes  Changes in staff attitudes and organizational culture  Conclusion: It would be particularly helpful for future research on EBCD to capture more robust data on the relative costs and benefits of implementing the approach compared to traditional patient involvement strategies and to provide greater insights what happens within the co-design phase of EBCD, particularly in terms of the diverse roles undertaken by patients and carers |
| Boivin  Involving patients in setting priorities for healthcare improvement: A cluster randomized trial  2014  Canada | Cluster randomised controlled trial | Primary healthcare  6 communities   - intervention (3 settings) - control (3 settings) | 83 chronic disease patients  89 healthcare professionals | Communities in a canadian region were required to set priorities for improving chronic disease management in primary care, from a list of 37 validated quality indicators  Intervention setting: patient involvement. Patients were consulted in writing, before participating in face-to-face deliberation with professionals  Control setting: no patient involvement | Primary outcome: the level of agreement between patients’ and professionals’ priorities  Secondary outcomes: healthcare professionals’ intention to use the selected quality indicators, and the costs of patient involvement  Priorities established with patients were more aligned with core generic components  41% increased agreement on common priorities  17% increased cost  10% increased time  Conclusions: Patient involvement can change priorities driving healthcare improvement at the population level. Future research should test the generalisability of these findings to other contexts, and assess its impact on patient care |
| de Souza  Patient involvement in rheumatology outpatient service design and delivery: a case study  2017  UK | Case study | Out-patient hospital care  The Group Rheumatology Initiative InvolvingPatients (GRIIP) project | RA patients  Patient experts  Healthcare professionals  Hospital management  Academics  10 project meetings | The GRIIP project; a joint venture between patients, clinicians, academics and management , with aim to develop patient-centred, innavotive strategies to improve the experience of RA patients.  The project resulted in three processes   - formation of an independent patient group (IPG) with 10 RA patients - a patient educational programme every 6 weeks - a mobile application | IPG:   - mean attendance 62% - several successful service improvements   Patient educational programme   - low mean attendance initially, but increased - positive experience by attendants   Mibile application   - downloaded 190 users - positive experience for users   Challenges and issues   - financial - lack of ongoing support from hospital management - poor attendance /punctuality at project meetings from healthcare professionals and management   Patients can actively shape services  Important role to drive change  Conclusion: Patients can effectively contribute to service improvement provided they are supported, respected as equals, and the organisation is willing to undergo a cultural change |
| Gustavsson  Improvements in neonatal care; using experience-based co-design.  2014  Sweden | Qualitative  Interviews | In-patient hospital care  Neonatal care | 5 parents  7 healthcare professionals | Eperience-based Co-design | Healthcare problems fall into simple, complicated and complex problems.  Staff and patient experiences with patient processes differ, and a collaborative approach is needed to capture all areas needing improvement. |
| Lachman  Developing person-centred analysis of harm in a paediatric hospital: a quality improvement report  2015  UK | Case study | In-patient paediatric hospital care  Renal ward | Patients and families  Healthcare professionals | Development and testing of a tool designed for patients and families to report harm   - questionnaire to families was tested in 20 Plan, Do, Study, Act (PDSA) cycles - design of the tool in cycles of PDSA, co-design and co-development - implementation of the tool | A simple, real-time bedside harm reporting tool for patients and their families   - families were successfully reporting incidents - families reported mostly miscommunication, medication, equipment use, significant delays to in-patient care, and problems related to cleanliness and hygiene - healthcare professionals’ reporting of critical incidents increased significantly by 67% - only 3% of the incidents reported by families were repeated by healthcare professionals - the tool facilitated active engagement with patients and families - the tool facilitated the opportunity for disclosure and learning for healthcare professionals - increased safety culture in the ward (although not significant change) |
| Lavoie-Tremblay  The perceptions of health care team members about engaging patients in care redesign  2014  Canada | Descriptive, qualitative study  Content analysis | In-hospital care  5 Transforming Care at the Bedside (TCAB) units in 3 hospitals | Managers  Healthcare professionals (73)  9 focus groups  13 individual interviews | A program called Transforming Care at the Bedside (TCAB), engaging patients as partners in care redesign teams  This study sought to explore the perceptions of healthcare professionals, and to examine the facilitating factors, barriers, and effects of such engagement | TCAB-teams benefit from involving patients in the care redesign process   - patients contribute to the decision-making process by adding information not previously thought about - facilitating factors, i.e. usage of various methods for communication, working with patients who had the same experiences, and open-mindness - barriers, i. e. physical and health limitations of the patient representatives - impact of patient engagement in TCAB, i.e. better informed in-patients with families and patients receiving better suited care - recommendations to involve patients earlier   Conclusion: Healthcare teams benefit from engaging patients in the change process. Patients contribute a different point of view, and this helps to ensure that the changes proposed and implemented address their needs |
| Locock  Using a national archive of patient experience narratives to promote local patient-centered quality improvement: An ethnographic process evaluation of ‘accelerated’ experience-based co-design  2014  UK | Ethnographic process evaluation  Mixed methods:   - quantitative - qualitative   Questionnaires  Observations  Interviews  Documentation | In-patient hospital care  2 hospitals   - intensive care - lung cancer care | 63 patients and family members  96 healthcare professionals | Accelerated Experience-based Co-design (AEBCD)  -local patient interviews were replaced with films from the national archive of patient experience narratives  48 co-design activities | 48 co-design activities  Cost reductions in the ‘discovery’ phase  AEBCD proved acceptable to patients and healthcare professionals  National films triggered discussion between patients’ and staff  More quickly achievement than EBCD  Lower cost than EBCD  Conclusions: Accelerated EBCD offers a rigorous and relatively cost-effective patient-centered quality improvement approach |
| Morrison  Beyond tokenistic participation: using representational artefacts to enable meaningful public participation in health service design  2013  UK | Case study  Participatory design | Out-patient services for older people | Public participants  Healthcare professionals  Participants in this project were involved in story telling, emotional maps and raod maps | The project Better Outpatient Services for Older People (BOSOP), a one year service improvement project  EBD  Three representational artefacts are described: emotion maps, stories, and tracing paper | Appropriate artefacts enables collaboration between public participants and health professionals in ways that both groups find meaningful and valid  Authors suggest that using such artefacts can provide an alternative approach to participation that stands in contrast to the current focus on the professionalisation of public participants  Conclusion: including participatory designers in projects, to chose or design appropriate representational arte- facts, can help to move beyond tokenistic participation in health service design, and address the policy–practice gap |
| Noergaard  Participatory action research in the field of neonatal intensive care: Developing an intervention to meet the fathers’ needs. A case study  2016  Denmark | Participatory action research  Grounded theory | In-patient hospital care  Neonatal intensive care  A 22-bed level 2 (≥28 weeks) NICU | 12 fathers  11 mothers  46 health professionals and managers | Participatory Action Research (PAR) to develop a father-friendly NICU where both the needs of fathers and mothers are met  PAR involves fathers, mothers, interdisciplinary healthcare professionals, and managers   - observations - semi-structured interviews - multi sequential interviews - workshops - focus groups - group discussion seminar   Data collection, analyses, and results were discussed with the participants | The paper describes and discusses participatory action research (PAR) as a method to improve NICUs’ service for fathers  In collaboration with the participants, the research team obtained knowledge and understanding of the fathers’ needs and wishes that contributed to an agreed concept of a father-friendly NICU  Conclusions: This paper contributed new knowledge of how PAR can be used to ensure that participants engaged in the field are involved in the entire process; consequently, this will ensure that the changes are feasible and sustainable |
| Olsson  Community collaboration to increase foreign-born women's participation in a cervical cancer screening program in Sweden: A quality improvement project.  2014  Sweden | Mixed methods:   - quantitative - qualitative   Focus group interviews  Content analysis | Primary care  Cervical cancer screening program | 13 doulas | Collaboration with local doulas to increase the cervical cancer screening program participation rate   - two focus group interviews with the doulas - Ishikawa diagram - control chart - introductory training from midwife - doulas making presentations at local events and association meetings - doulas and midwives working together when presenting and in the use of a mobile unit for Pap smear testing | Identification of barriers that prevent foreign-born women from participating in the cervical cancer screening program  After one year, the number of Pap smear tests in the area had increased by 42%  Conclusions: This paper suggests that several barriers make participation in cervical cancer screening program more difficult for foreign-born women in Sweden. Specifically, these barriers include lack of knowledge concerning cancer and the importance of preventive healthcare services and practical obstacles such as unavailable child care and language skills. The overarching approach to surmount these barriers was to engage persons with a shared cultural background and mother tongue as the target audience to verbally communicate information. The doulas who helped to identify barriers and plan and execute interventions gained increased confidence and a sense of pride in assisting to bridge the gap between healthcare providers and users |
| Pittens  The involvement of gynaecological patients in the development of a clinical guideline for resumption of (work) activities in the Netherlands  2015  The Netherlands | Case study  Evaluation framework | In-hospital care  Obstetrics and gynaecology  The EMGO Institute for Health and Care Research | Patients  Healthcare professionals  3 focus group discussions (FGD) with 21 participants  20 participants of the agreed to test the web-based patient version  15 participants completed a questionnaire regarding feasibility, content and design | The development of a clinical guideline for resumption of (work) activities  Process:  At three different stages patients were involved in the process:   - FGD - patients were involved for the instruction video - patients tested the patient version of the clinical guideline | 1. A clinical guideline with recommendations for resumption of (work) activities after gynaecological surgery 2. A web-based patient version of the clinical guideline   Description of patient involvement in the development process   - experiential knowledge of patients vs. the expert knowledge of healthcare professionals - there was no interaction between patients and healthcare professionals, which prevented optimal mutual learning - the diversity among participants stimulated co-creation of solutions   Conclusions: Consultation of individual patients by means of FGDs and with regular feedback moments has been rather effective for a guideline development process related to an incidental, non-threatening disease for which there is no patient organisation. Patients’ input contributed to applicability of the clinical guideline in daily practice. Increased patient involvement could be achieved by integration of the two parallel trajectories with additional participatory activities, such as a dialogue meeting |
| Rise  Experiences from the implementation of a comprehensive development plan for user involvement in a mental health hospital: a qualitative case study  2014  Norway | Case study  Qualitative    Semi-structured interviews  Observations | In-hospital care  Mental health | 4 user representatives  13 healthcare professionals  10 meetings | Implementation of a comprehensive development plan intended to enhance user involvement in a mental health hospital | Three different stories on implementation experience   - implementation as success - implementation with obstacles - development plan had limited impact   The implementation was described differently depending on the participants’ position in the organisation  Conclusions: Close attention should be made to decision-making and resource allocation when implementing user involvement |
| Robben  Filling the gaps in a fragmented health care system: development of the Health and Welfare Information Portal (ZWIP)  2012  The Netherlands | Case study  Interviews  Working groups | Out-patient care  Healthcare and welfare services  Frail people | Collaboration of stakeholders   - patients - informal caregivers - healthcare professionals | Development of the Health and Welfare Information Portal (ZWIP), a personal, internet-based conference table for multidisciplinary communication and information exchange for patients, their informal caregivers and healthcare professionals  The intervention mapping process:   1. needs assessment 2. preparing matrices of performance objectives and determinants 3. selecting theory-informed intervention methods and practical strategies 4. producing program components and materials 5. planning program adoption, implementation, and sustainability 6. planning for evaluation | The Health and Welfare Information Portal (ZWIP)  Conclusions: This paper describes the successful development and the content for the ZWIP as well as the strategies developed for its implementation. Throughout the development, representatives of future users were involved extensively. Future studies will establish the effects of the ZWIP on self-management and shared decision making by frail older people as well as on collaboration among the professionals involved |
| Tollyfield  Facilitating an accelerated experience-based co-design project  2014  UK | Case study  Qualitative  Interviews  Feedback forms | In-hospital care  Adult critical care | 19 patients and carers  50 healthcare professionals (15 interviewed) | Accelerated Experience-based Co-design (AEBCD) | The importance of   - training and support for facilitators - engaging healthcare professionals and patients - planning and organisation - facilitation of events and co-design group meetings   Patients, carers and healthcare professionals agreed on four key priorities that generated 29 action points for the 4 co-design teams  A very positive experience for patients, carers, and healthcare professionals  The AEBCD approach is spread to other departments  Conclusion: The importance of engaging with patients, and their relatives, and seeking their opinion and feedback has been widely publicized in the literature. It is usually undertaken via comments cards and questionnaires yet the AEBCD process and this project took the next step forward. It truly engaged with patients and their carers. Staff also had the opportunity to express their opinions, and staff, patients and carers alike were provided with a forum in which they could work together to generate service improvements for all |
| Tsianakas  Implementing patient-centred cancer care: using experience-based co-design to improve patient experience in breast and lung cancer services  2012  UK | Case study  Ethnographic observation  Qualitative  Interviews  Observations | In-hospital care   - breast cancer - lung cancer | 36 patients  (5 interviewed)  63 healthcare professionals  (4 interviewed)  36 filmed narrative patient interviews  219 hours of ethnographic observation | Experience-based Co-design (EBCD) | Patients from both tumour groups identified similar issues  The QI priorities patients and healthcare professionals chose to work on together were tumour specific  Increased patient involvement, responsibility and empowerment  A sense of community  A close connection between experiences and subsequent improvement priorities  Conclusion: EBCD positions patients as active partners with healthcare professionals in QI. Breast and lung cancer patients identified similar touchpoints in their experiences, but these were translated into different improvement priorities for each tumour type. This is an important consideration when developing patient-centred cancer services across different tumour types |
| Worswick  Interprofessional learning in primary care: an exploration of the service user experience leads to a new model for co-learning.  2015  UK | Mixed methods:   - quantitative - qualitative   Data from LIMBIC project  Semi-structured interviews | Primary healthcare  Back pain care | Service users (9 general practice teams)  11 patient representatives  Focus group transcripts  Patient stories  Film  E-mails  Meeting notes  Educational material  Presentations | An interprofessional educational initiative, the Learning to Improve the Management of Pain in the Community (LIMBIC) project   - workshops - QI projects | A model for co-learning with service users  Results illustrated the importance, to the service users, of the sense of community, of clear communication, and of influencing change through involvement  Conclusions: The experience of service users can be optimized by planning, preparation and support so that their wealth of expertise can be recognized and utilized. A model for co-learning was developed and is presented in this paper |
