# Supplementary material for: How might patient involvement in healthcare quality improvement efforts work—A realist literature review
Source: Health Expect. 2019 May 1;22(5):952–64. doi: 10.1111/hex.12900 (PMC6803394; doi:10.1111/hex.12900)
Supplement: Supplementary file 2 [file HEX-22-952-s002.docx]

**Supplement 2.** Examples of how CMO-configurations, ‘middle-range theories’ and theories were derived from the data.

| Article | **C-M-O-configuration**  ‘M resource + C → M reasoning = O’ | **Middle-range theories** | **Thematic organising**   1. Key mechanisms 2. Contextual factors 3. The role of the healthcare organisation | **Theories** | **Quotations** |
| --- | --- | --- | --- | --- | --- |
| Locock L, Robert G, Boaz A, Vougioukalou S, Shuldham C, Fielden J, *et al*. Using a national archive of patient experience narratives to promote local patient-centered quality improvement: an ethnographic process evaluation of ‘accelerated’ experience-based co-design. *Journal of Health Services Research & Policy,* 2014; 19(4): 200-207. | **M** Accelerated Experience-Based Co-Design using relevant archived films and face-to-face meetings of local patients and clinicians  **+ C** Multi-professional clinical teams and their patients who have had hospital care in NHS England  **→ M** Understanding patient and clinician experiences and opportunities for improvement  **= O** Collaborative efforts to improve care. | Triggering discussion and facilitating interaction for staff and patients in physical meetings leads to increased understanding and collaborative efforts to improve care. | 1. Interaction possibilities and mutual trust at the microsystem level. Mutual understanding from patient and healthcare professionals experiences raise opportunities for co-design improvement work.  2. Collaborative improvement work.  3. Facilitating co-design improvement work at the microsystem level. | **1:** Tailor user involvement to the various QI efforts and contexts.  **2:** Support interaction and partnership within each specific QI effort.  **3:** Support the behavioural change, that follows from QI efforts involving users, at all levels of the organisation. | “…our observations suggest face-to-face encounters with patients have been even more transformative, inspiring and revelatory to staff in making them think differently about their values and practice. Having continued patient involvement helps ensure improvements really do address patient concerns and holds staff to account to see change through. Patients’ physical presence constantly reminds everyone who change is for, and why it matters compared to other potentially overwhelming work pressures and demands… Patients, too, report a new level of appreciation for staff, a belief that they will be listened to and that change is possible, and renewed trust in their local [health system]." |
| Tollyfield R. Facilitating an accelerated experience-based co-design project. *British Journal of Nursing,* 2014; 23(3): 136-141. | **M** AEBCD (Accelerated Experience-based Co-design) **+ C** in-hospital care; cardiothoracic intensive therapy unit **→ M** organisational reasoning: support from management, staff engagement and facilitator with insight and experience of context **= O** thorough pre-planning and preparation of each event and co-design group and proactivity  **M** Thorough pre-planning and preparation of each event and co-design group and proactivity **+ C** in-hospital care; cardiothoracic intensive therapy unit **→ M** by mutual respect and equal partnership in welcoming, relaxed, clean and comfortable environment **= O** reflection in co-design groups, participants listening to each other  **M** reflection in co-design groups, participants listening to each other  **+ C** in-hospital care; cardiothoracic intensive therapy unit **→ M** through development of new relationships and ways of working together **= O** Intervention: 4 key priorities and 29 action points Study of intervention: Initial apprehension; a very positive experience for both staff, patients and carers. | Accelerated Experience-based Co-design (AEBCD) in in-hospital care works well with organisational support from management and facilitator with insight and experience of context.  Mutual respect and equal partnership in welcoming, relaxed, clean and comfortable environment encourages reflection in co-design groups and participants listening to each other, and promotes the development of new relationships and ways of working together. | 1. Mutual trust at the microsystem level  2. Interaction possibilities for patients and healthcare professionals and organisational support in improvement project  3. Interaction possibilities for patients and healthcare professionals in improvement project  4. Organisational support in co-design improvement projects | **1:** Tailor user involvement to the various QI efforts and contexts.  **2:** Support interaction and partnership within each specific QI effort.  **3:** Support the behavioural change, that follows from QI efforts involving users, at all levels of the organisation. | "…listening to patients' experiences and designing services with them ensures that services are truly patient-centred."  "All participants really listened to each other, as if for the first time, and got to know and understand each other as individuals as well as professionals while developing new patient, carer and staff relationships and ways of working together."  "Staff were given the opportunity to reconnect with their core values of caring and compassion."  "...staff ...also had the opportunity to express their opinions..."  "...staff, patients and carers alike were provided with a forum in which they could work together to generate service improvements for all." |
| de Souza S, Galloway J, Simpson C, Chura R, Dobson J, Gullik NJ, *et al.* Patient involvement in rheumatology outpatient service design and delivery: a case study. *Health Expectations,* 2016; 20: 508-518. | **M (resource)** Patient involvement through an independent patient group at hospital organisational level **+ C** rheumatology outpatient services **→ M (reasoning)** Dedicated and committed patients and clinicians **= O s**everal successful service improvements **= O** development of patient educational evenings **= O** development of mobile application  **M (resource)** Patient involvement through an independent patient group at hospital organisational level **+ C** rheumatology outpatient services **→ M (reasoning)** lack of organisational support **→ M (reasoning)** lack of time and commitment from clinicians and management **= O** delays in starting and fulfilling the project  **= O** IPE experiencing frustration | The degree and success of patient involvement depends on organisational support and if there is commitment from patients and healthcare professionals. | 1. Patient involvement through an independent patient group at hospital organisational level  2.The degree of dedication and commitment (positive or negative) of patients and clinicians  3.Dedicated and committed patients and clinicians influence success in healthcare service improvements  4. Organisational support to prevent barriers related to time commitment, organisational culture (the belief by clinicians and managers that patients cannot make an effective contribution) and the perceived threat to organisations of 'losing face' by sharing their organisational shortcomings and difficulties. | **1:** Tailor user involvement to the various QI efforts and contexts.  **2:** Support interaction and partnership within each specific QI effort.  **3:** Support the behavioural change, that follows from QI efforts involving users, at all levels of the organisation. | "The process allowed patients to directly contribute to shaping the services they receive long-term and to realize their options were of value to clinical staff and hospital management."  "Initially, the feedback mechanism was more formal, but now that relationships have been established between patient experts, clinicians and management; patient experts can informally and directly raise clinic issues..."  "... the extent to which involved patients influence service design and improvements seems to be of greater importance than their perceived 'representativeness'. |
| Noergaard B, Johannessen H, Fenger-Gron J, Kofoed P-E, Ammentorp J. Participatory action research in the field of neonatal intensive care: developing an intervention to meet the fathers’ needs. A case study. *Journal of Public Health Research,* 2016; 5(744): 122-129. | **M (resource)** A PAR project to develop a father-friendly NICU  **+ C** NICU **→ M (reasoning)** involving the adequate stakeholders **= O**  open communication and collaboration **= O** respectful, non-judgmental and permissive context  **M (resource)** open communication and collaboration **M (resource)** respectful, non-judgmental and permissive context  **+ C** NICU **→ M (reasoning)** activities that encourage participants to share experiences and prime new ideas **= O** changes based on participants' experiences   **M (resource)** changes based on participants' experiences  **+ C** NICU **→ M (reasoning)** respectful, non-judgmental and permissive context  **= O** possibility for feasible and sustainable changes | PAR in a care redesign project supports personalised collaboration between patients and healthcare professionals and can lead to sustainable organisational and attitudinal changes in both patients and healthcare professionals. | 1. Open communication and collaboration in a respectful, non-judgmental and permissive context  2. A PAR project design involving the adequate stakeholders    3. The PAR activities that encourage participants to share experiences and prime new ideas  4. Facilitating interactions between participants in a respectful, non-judgmental and permissive context, and facilitating changes based on participants' experiences | **1:** Tailor user involvement to the various QI efforts and contexts.  **2:** Support interaction and partnership within each specific QI effort.  **3:** Support the behavioural change, that follows from QI efforts involving users, at all levels of the organisation. | "In collaboration with the participants, the research team obtained knowledge and understanding of the fathers' needs and wishes that contributed to the concept of a father-friendly NICU."  "By engaging the stakeholders in the process, the culture of the NICU became open to reflection and action."  "By using the right activities in the right context, we gained an opportunity to promote participants' creativity."  "This small case study may have implications on how PAR can be employed to ensure that changes, which will help develop a more father-friendly NICU, are based on participants' experiences and are feasible and sustainable." |
| Pittens CACM, Vonk Noordegraaf A, van Veen S, Anema JR, Huirne JAF, Broerse JEW. The involvement of gynaecological patients in the development of a clinical guideline for resumption of (work) activities in the Netherlands. *Health Expectations,* 2013; 18: 1397-1412. | **M (resource)** Patient involvement in the development of a web-based patient version of the clinical guideline **+ C** gynaecological patients **→ M (reasoning)** applying focus group technique in a comfortable environment **= O** co-construction of meaning and understanding **= O** substantial contribution of patients  **M (resource)** Patient contribution in the development of a web-based patient version of the clinical guideline **+ C** gynaecological patients **→ M (reasoning)** project leaders sharing patient experiences with healthcare professionals **= O** patients indirectly contributing to the formulation of some topics for the professionals development of clinical guideline recommendations  **M (resource)** Patient involvement in the development of a web-based patient version of the clinical guideline **+ C** gynaecological patients **→ M (reasoning)** no facilitation of direct interaction between patients and professionals   **= O** mutual learning and development did not occur | Patient involvement in the development of the recommendations of clinical guidelines may result in increased relevance and quality of the recommendations.  No facilitation of direct interaction between patients and professionals  prevents mutual learning and development. | 1. Patient involvement in the development of a web-based patient version of the clinical guideline  2. There was no facilitation of direct interaction between patients and professionals leading to patients only indirectly contributing and the power of decision making remaining with the professionals. Mutual learning and development did not occur.  3. Project leaders acting as intermediators between patients and healthcare professionals did facilitate to some extent.  4. Facilitating interactions between participants in a respectful, non-judgmental and permissive context, and facilitating changes based on participants' experiences. | **1:** Tailor user involvement to the various QI efforts and contexts.  **2:** Support interaction and partnership within each specific QI effort.  **3:** Support the behavioural change, that follows from QI efforts involving users, at all levels of the organisation. | "Patients and professionals were not equally involved. Patients were involved on the level of consultation, while professionals were involved on a higher level. By their participation in the Delphi study, professionals had decision-making power about the recommendations for resumption of (work) activities."  "They (researcher and project leaders) believed specific medical and professional knowledge were required for the formulation and interpretation of the recommendations. As a consequence, the involvement of patients was mainly restricted to the web-based patient version. The contribution of the representative of the umbrella organization was appreciated by the other members of the advisory committee. They considered her as equal and professional."  "Patients were able to participate in this clinical guideline development process, because their involvement did not require specific skills; only their experiential knowledge was addressed."  "Although the followed approach turned out to be quite successful, one could question to what extent a more interactive process would have had an added value on the quality of the recommendations."  "... patients' input complemented the input of professionals and increased the applicability of the recommendations in daily practice." |
